# Extremely Monodispersed Micrometer-Scale Spherical Particle Synthesis of Ag Inside a Microdroplet Vaporizing in Plasma

**DOI:** 10.1021/acsomega.3c10215

**Published:** 2024-03-12

**Authors:** Kaishu Nitta, Takeru Hato, Hitoshi Muneoka, Yoshiki Shimizu, Kazuo Terashima, Tsuyohito Ito

**Affiliations:** † Department of Advanced Materials Science, Graduate School of Frontier Sciences, The University of Tokyo, 5-1-5 Kashiwanoha, Kashiwa, Chiba 277-8561, Japan; ‡ AIST-UTokyo Advanced Operando-Measurement Technology Open Innovation Laboratory (OPERANDO-OIL), National Institute of Advanced Industrial Science and Technology (AIST), 5-1-5 Kashiwanoha, Kashiwa, Chiba 277-8589, Japan

## Abstract

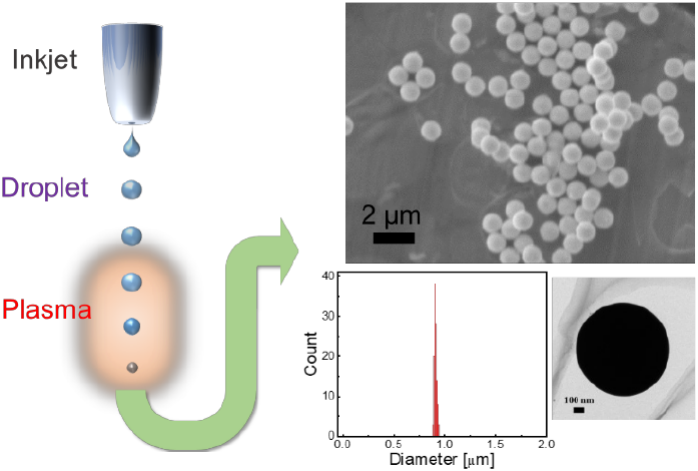

Spherical Ag particles have received considerable attention because of their unique properties as well as their applications in various fields. In the present study, the synthesis of micrometer-scale spherical Ag particles with an extremely narrow size distribution is demonstrated using a simple capacitively coupled atmospheric-pressure plasma reactor with an inkjet head. Droplets of a Ag nitrate aqueous solution are ejected from the inkjet head to synthesize Ag particles. The gaseous temperature in the reactor is adjusted such that Ag can be melted with a negligibly small vapor pressure. These particles exhibit a spherical shape with a smooth surface. The mean diameter of the particles is 0.91 ± 0.013 μm with a small coefficient of variation of 1.5%, the smallest value ever reported for Ag particles of less than 1 μm. The grain sizes of the particles are larger than 100 nm, as expected from the broadening of the X-ray diffraction peaks. The excellent monodispersity of the particles synthesized by this method may expand the applications with micrometer-scale spheres such as ball spacer, microsized ball bearing, and inks for printed electronics.

## 1 Introduction

Spherical particles of crystalline inorganic materials, such as metals, oxides, carbides, and alloys, have attracted considerable interest for applications in optics, catalysis, biophotonics, and analytical chemistry.^1,2^ The monodispersibility and controllability of particle size are important factors in achieving optimum performance, because particle size affects electrical, optical, magnetic, thermoelectric, and photoelectric properties of materials.^3−5^ Micrometer-scale spheres, herein defined as spherical particles with dimeters of 0.1–5 μm, might be useful for electrical and optical applications in part because of their better dispersibility and higher light scattering efficiency than those of nanoparticles.^6,7^ Such micrometer-scale spheres, especially in the submicron size range, are useful in the medical field because of their unique capability to selectively enter tumor cells.^8^ Therefore, the synthesis of micrometer-scale spheres has recently assumed significant and urgency owing to increasing concerns regarding the evaluation of nanosize-dependent biotoxicity.^9^

Particle synthesis using top–down processes, such as milling, is gradually making it possible to create particles smaller than 1 μm with advances in technology;^10,11^ however, mechanical methods of crushing yield particles with nonsmooth angular surfaces. In contrast, the bottom–up process of growing particles from atoms or molecules, such as the chemical reduction method, produces uniform-size particles through uniform nucleation from the raw material solution and reaction time control.^12,13^ However, as the particle size increases, stable crystal planes tend to grow anisotropically, making it difficult to synthesize spheres with diameters larger than the submicron.^14^ Thus, the synthesis of crystalline material spheres, particularly those with smooth surfaces of the order of microns, is technically difficult and limited to a few methods, such as the laser melting method.^8,15−17^ Furthermore, it remains challenging to develop a method for directly synthesizing uniformly sized particles with a coefficient of variation (CV), which is the ratio of the standard deviation to the mean, of less than 5%.

In this study, we present a unique method for producing micrometer-scale spherical Ag particles with an extremely narrow size distribution via atmospheric-pressure plasma processing with inkjet droplets. In this process, droplets that are several tens of micrometers in diameter containing dissolved raw materials are ejected from an inkjet device and introduced directly into an atmospheric-pressure plasma. The ultrahigh-frequency atmospheric-pressure plasma reactor used in this study can easily generate a high-temperature reaction field with highly energetically charged particles and highly reactive radicals.^18^ The atmospheric-pressure plasma system thereby can produce the desired particles directly and rapidly (e.g., several to several tens of milliseconds).^3,19,20^ Furthermore, inkjet droplets have an extremely narrow size distribution (CV < 0.6%).^21^ Consequently, the synthesized particles have excellent reproducibility.^3,19^ In the present study, the environmental temperature was proactively controlled by pulse modulation of the applied power to generate an optimum environmental temperature that enabled the melting of Ag without evaporation in the plasma, thus achieving excellent reproducibility of the synthesized particles. Metallic particles with excellent monodispersibility may facilitate the development of applications such as ball spacer,^22^ microsized ball bearing,^23,24^ and inks for printed electronics,^25^ as well as electrical, optical, and medical applications. Furthermore, this method can directly deposit particles from an inkjet nozzle onto a substrate, and the ability to adjust the adhesion position of synthesized particles on the spot may expand their application possibilities.

## 2 Results and Discussion

**Figure 1:**
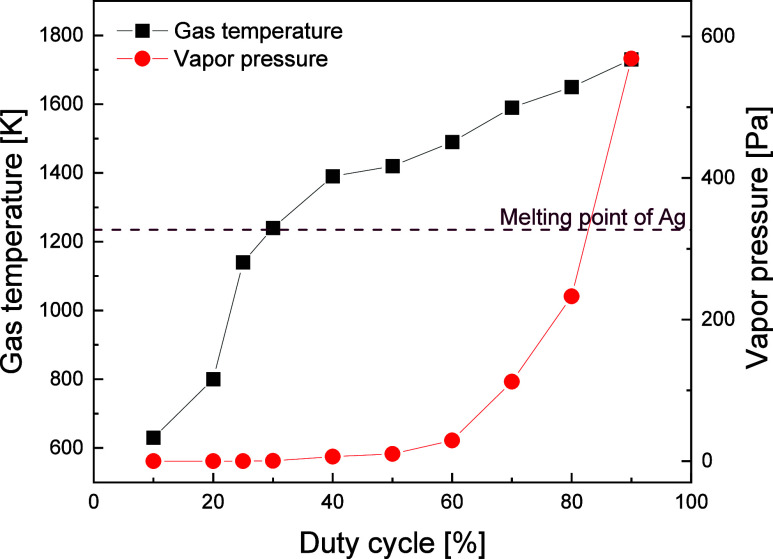
Estimated plasma gas temperature and corresponding Ag vapor pressure at the gas temperature as functions of the duty cycle of plasma-generation power.

Figure 1 depicts the variation in plasma gas temperature, which is estimated from the OH optical emission spectra at approximately 310 nm via a spectral fitting technique using two-temperature analysis^26,27^ as a function of the duty cycle of the plasma-generation power. The vapor pressures of Ag at the equilibrium temperature with the estimated gas temperature are also plotted, referring to the literature by Panish.^28^ In this study, the duty cycle and input power of the plasma generation were adjusted to 50% and 50 W, respectively, to elevate the temperature inside the reactor to approximately 1420 K during particle synthesis. At this temperature, Ag can melt with negligible vapor pressure (∼10 Pa).^28^ Most Ag atoms are expected to remain in the final particle without evaporation, but a spherical shape can be achieved after melting. Thus, a microdroplet should function as a semiclosed microreactor under this condition with a low vapor pressure.

**Figure 2:**
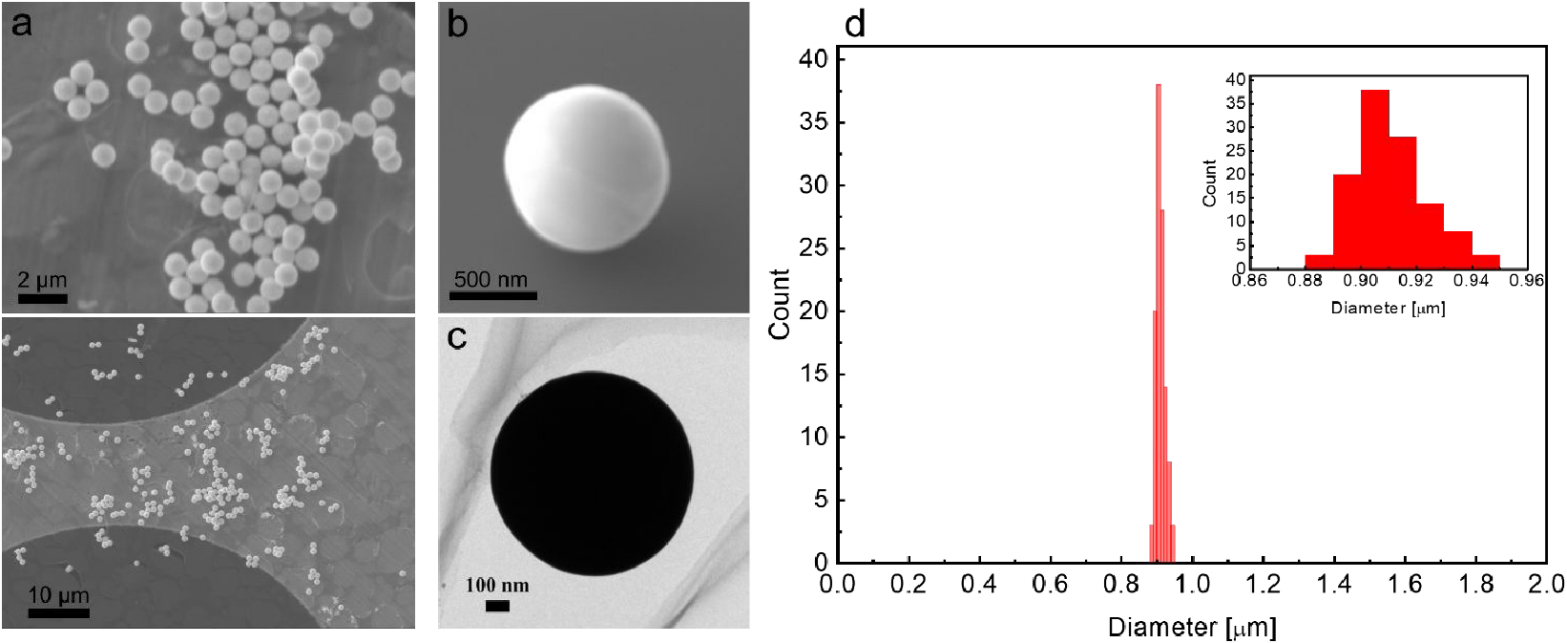
(A) SEM, (b) FE-SEM, and (c) TEM images of synthesized particles. (d) Particle size distribution of the synthesized particles.

Figure 2a–c shows the scanning electron microscope (SEM), field emission-SEM (FE-SEM), and transmission electron microscope (TEM) images of particles synthesized using an Ag nitrate (AgNO_3_) solution at a concentration of 10 mM. All of the particles had clear spherical shapes with almost identical diameters and exhibited a high degree of smoothness. Figure 2d shows the size distribution of the particles on a silicon wafer analyzed using SEM images. The mean and standard deviation of the diameter of 114 particles were 0.91 and 0.013 μm, respectively, resulting in a CV value of 1.5%. The CV of the synthesized Ag particles was significantly smaller than that of Au particles synthesized from similar inkjet droplets in a previous study (3–9%),^3^ where the synthesis environment temperature (approximately 1080 K) was lower than the melting point of Au (approximately 1337 K). The average circularity (perimeter ratio) obtained from the particle area projections depicted in the SEM images using the formula proposed by Cox^29^ was 0.95 ± 0.01, comparable to that of previously reported Au particles (0.75–0.95).^3^ In this study, the environmental temperature (approximately 1420 K) was adjusted to exceed the melting point of Ag (approximately 1235 K). Therefore, during the flight in the plasma after complete solvent evaporation, the precipitated fine Ag crystals melted, filled the voids between the crystals, grew into larger crystals, and formed spherical micrometer-scale particles because of surface tension, resulting in the synthesis of particles with more uniform size and excellent sphericity. Assuming that the dissolved Ag atoms in one droplet (whose diameter was measured to be approximately 20 μm using a charge-coupled-device (CCD) camera (Watec, WAT-902H ULTIMATE)) are stored in one synthesized particle to form a perfectly spherical dense Ag particle, the estimated particle size is approximately 0.93 μm. This size is close to the experimentally observed particle sizes and supports the idea that the microdroplet acts as a semiclosed microreactor, even when considering errors in the droplet size measurement, solution conditioning, and SEM image analysis. Since no particles that appear to be in the middle of the reaction have been observed, it seems that each droplet has sufficiently reacted to form the final Ag particles. However, the current method of depositing particles directly onto Si wafers may not provide 100% particle collection efficiency. In the future, by improving the collection efficiency using methods such as electrostatic precipitation, it may become possible to precisely control the number of obtained particles with the control of the number of ejected droplets. It may also be possible to control the final particle size by adjusting the initial solution concentration, as reported for the synthesis of Au particles in the range of 0.3–0.6 μm.^3^

**Figure 3:**
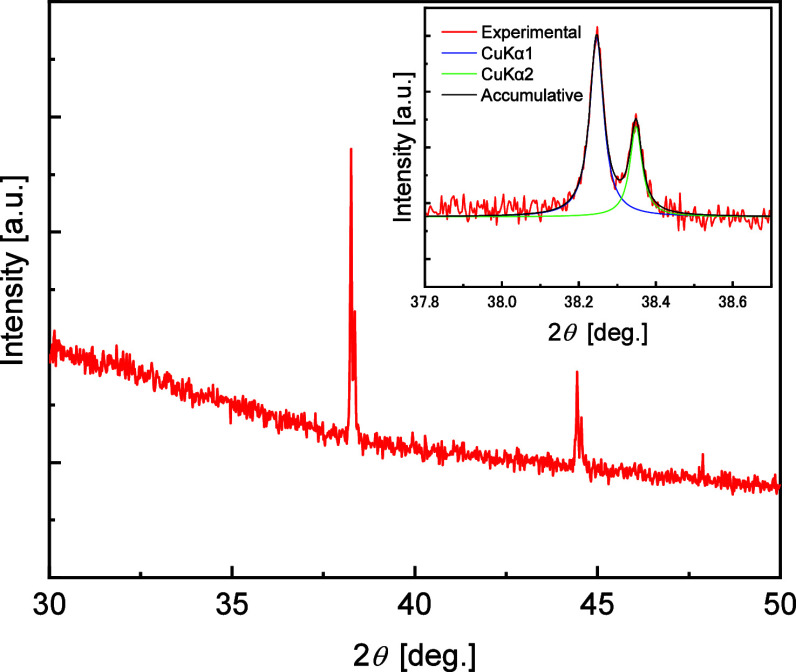
XRD pattern of synthesized particles on a silicon substrate. Laurentian fitting result of diffraction peaks by CuKα1 and CuKα2 rays around 38.3° is presented in the upper right panel.

Figure 3 shows the X-ray diffraction (XRD) patterns of the particles synthesized on a silicon substrate. The peaks at approximately 38.3° and 44.4°, which were derived from the (1 1 1) and (2 0 0) planes, respectively, correspond to Ag (Powder Diffraction File (PDF) card # 00-004-0783). This result clearly indicates that a certain amount of crystalline Ag is present in the particles. As shown in the upper right panel in Figure 3, the diffraction peaks by CuKα1 and CuKα2 rays on Ag (1 1 1) planes can be observed (also seen in the diffraction peaks around 44.4°); the peak shapes were fitted by the Lorentzian function, and the broadening width of each diffraction peak was measured. When the crystallite size of the synthetic material is of the nanoscale (less than 100–200 nm), the average crystallite size can be estimated from the broadening of the XRD peaks using the Scherrer equation.^30,31^ However, in the present case, the broadening of each Ag diffraction peak (full width at half-maximum: 0.04°) was no broader than that of the instrumental broadening, which was measured for the bulk Ag and alumina reference samples. This result suggests that the crystallite size of the synthesized Ag particles is larger than 100 nm. Given that the average particle size is 0.91 μm, the synthetic particles are likely composed of several crystallites or single crystals.

**Figure 4:**
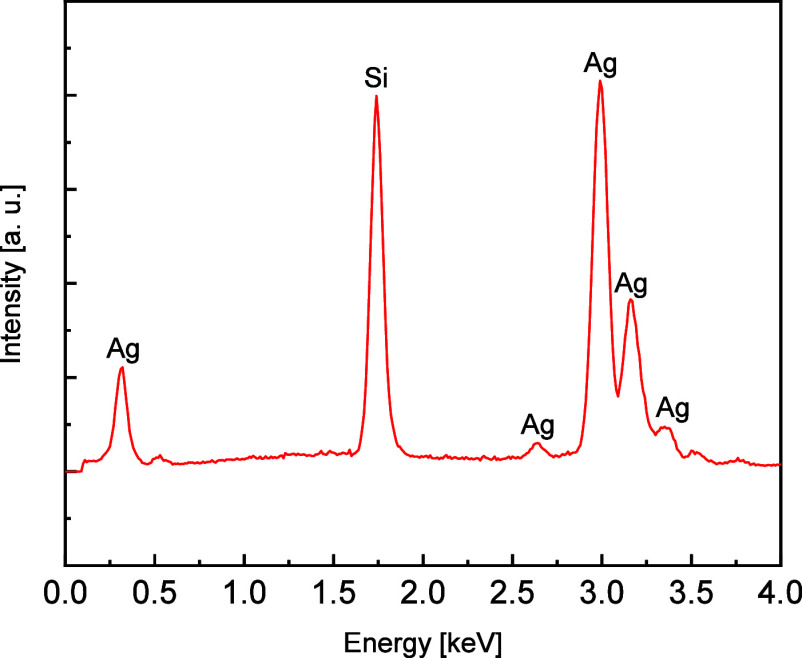
EDS spectra of synthesized particles on a silicon substrate.

Figure 4 shows the energy-dispersive X-ray spectroscopy (EDS) spectra of the synthesized particles. Ag atoms in the particles were identified as well as Si atoms that may have originated from the substrate. The XRD and EDS characterizations of as-prepared Ag spheres suggest that the resulting products mostly contain crystal Ag.

**Figure 5:**
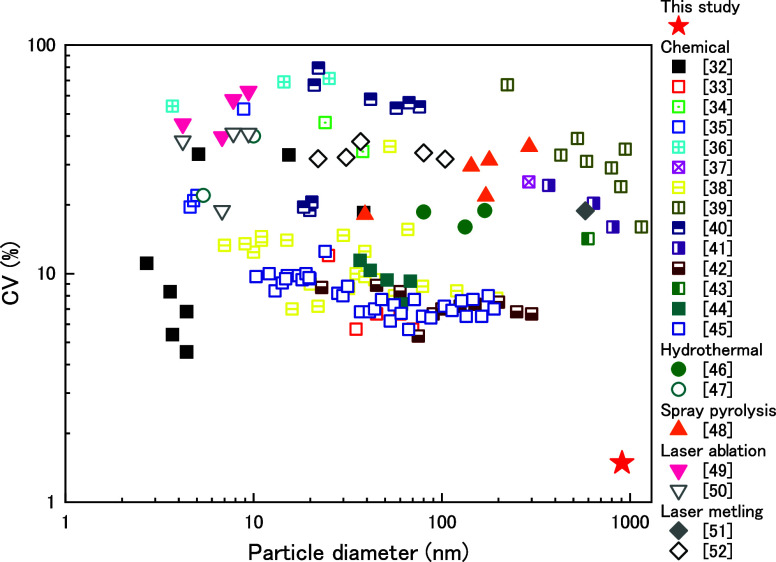
Comparison of CV values and particle diameters of synthesized particles with those reported in literature for spherical or quasi-spherical Ag particles using various particle synthesis methods.^32−52^

Figure 5 compares our CV value with previously reported values obtained using various particle synthesis methods, such as chemical reduction,^32−45^ hydrothermal synthesis,^46,47^ spray pyrolysis,^48^ laser ablation,^49,50^ and laser melting.^51,52^ For relatively small particle sizes (less than 300 nm), monodisperse particles with CV values less than 10% can be synthesized by using chemical reduction methods. For relatively large particle sizes (measuring micrometers), there are few reports of CV values below 10%. Therefore, our reported CV value of 1.5% for a size near 1 μm is extremely small compared to those reported in previous studies. Furthermore, reported examples of spherical Ag particles larger than a few hundred nanometers are often aggregates of nanocrystals and tend to have a relatively rugged surface.^4,39,41,53^

## 3 Conclusions

In conclusion, an innovative approach for preparing micrometer-scale Ag spheres with an extremely small CV of 1.5% via atmospheric-pressure plasma processing with inkjet droplets was proposed in this study. Inkjet droplets with negligibly small size variations were used as microreactors and reacted in atmospheric-pressure plasma at a controlled ambient temperature to rapidly form Ag spheres in a single step. The simplicity of the proposed method makes it an advantageous route for manufacturing micrometer-scale spheres for various applications.

## 4 Experimental Section

### 4.1 Materials

AgNO_3_ and deionized water were purchased from Fujifilm Wako Chemical Ltd. and mixed to prepare a 10 mM solution. Ar (>99.9995%) as discharge gas was purchased from Taiyo Nippon Sanso Corp.

### 4.2 Sample Preparation

A capacitively coupled atmospheric-pressure plasma reactor with an inkjet head was used for particle synthesis. The details of the experimental setup are reported elsewhere.^3^ In this study, the discharge gap was 1 mm, and vertical and depth lengths of the parallel plates formed by the pair of copper electrodes were 10 and 2 mm, respectively. For plasma generation, an ultrahigh-frequency wave of 450 MHz with pulse modulation (duty cycle: 50%, frequency: 100 kHz, input power: 50 W) was applied using a power supply (Tokyo Hi-power, RF50–450-P). The Ar gas flow rate was set to 30 sccm. Droplets of the Ag nitrate aqueous solution were ejected from the inkjet head (Microjet, IJHD-10) at a frequency of 100 Hz. The residence time of the droplets or synthesized particles in the plasma, estimated based on gas velocity, assuming an ideal gas condition,^18^ was approximately 8 ms. The synthesized particles were deposited on a p-type silicon wafer (Nilaco Corp.). For TEM observations, some of the particles on the silicon wafer were transferred to a TEM grid (Okenshoji Co., Ltd.).

### 4.3 Characterization

The particles on the Si wafer or TEM grid were observed using scanning electron microscope SEM (JEOL, JSM-IT500), FE-SEM (JEOL, JIB-4700F), and TEM (JEOL, JEM-2100). The SEM images were analyzed using MATLAB (Mathworks, Inc., Natick, MA, USA) to determine the particle size distribution. The crystalline nature of the samples was analyzed by using XRD (Rigaku, Smartlab BBKC). The atomic composition of the particles was determined using EDS equipped with SEM (JEOL, JSM-IT500). Optical emission spectroscopy of the plasma was performed by using an intensified CCD camera (Hamamatsu Photonics, C8484-05G01 and C7164-03) equipped with a spectrometer (HORIBA Scientific, iHR320).
